# Results of robotic liver surgery in association with IWATE criteria — the first 100 cases

**DOI:** 10.1007/s00423-024-03239-6

**Published:** 2024-02-02

**Authors:** Kira C. Steinkraus, Benno Traub, Patrick Heger, Marin Zaimi, Andre L. Mihaljevic, Christoph W. Michalski, Marko Kornmann, Felix J. Hüttner

**Affiliations:** 1 Department of General and Visceral Surgery, Ulm University Hospital, Ulm, Germany; 2 Present Address: Department of General, Visceral and Transplantation Surgery, Tübingen University Hospital, Tubingen, Germany; 3 Present Address: Department of General, Visceral and Transplantation Surgery, Heidelberg University Hospital, Heidelberg, Germany; 4 Present Address: Department of General, Visceral and Thoracic Surgery, Klinikum Nürnberg, Paracelsus Medical University, Nurnberg, Germany

**Keywords:** Robotic liver surgery, IWATE criteria, Surgical site infections

## Abstract

**Background:**

Aim of the current study was to present the results of the implementation phase of a robotic liver surgery program and to assess the validity of the IWATE difficulty score in predicting difficulty and postoperative complications in robotic liver surgery.

**Methods:**

Based on the prospective database of the Interdisciplinary Robotic Center of Ulm University Hospital, the first 100 robotic liver surgeries were identified and analyzed. Perioperative parameters (duration of surgery and blood loss) and postoperative parameters including morbidity, mortality, and length of hospital stay were assessed and the results were compared between different IWATE difficulty categories.

**Results:**

From November 2020 until January 2023, 100 robotic liver surgeries were performed (41 female, 59 male; median age 60.6 years, median BMI 25.9 kg/m^2^). Median duration of surgery was 180 min (IQR: 128.7), and median blood loss was 300 ml (IQR: 550). Ninety-day mortality was 2%, and overall morbidity was 21%, with major complications occurring in 13% of patients (≥ grade 3 according to Clavien/Dindo). A clinically relevant postoperative biliary leakage was observed in 3 patients. Posthepatectomy liver failure occurred in 7% (4 Grade A, 3 Grade B). Duration of surgery (*p* < 0.001), blood loss (*p* < 0.001), CCI (*p* = 0.004), overall morbidity (*p* = 0.004), and length of hospital stay (*p* < 0.001) were significantly increased in the IWATE ‘expert’ category compared to lower categories.

**Discussion:**

Robotic surgery offers a minimally invasive approach for liver surgery with favorable clinical outcomes, even in the implementation phase. In the current study the IWATE difficulty score had the ability to predict both difficulty of surgery as well as postoperative outcomes when assessing the complexity of robotic liver surgery. Therefore, the role of the IWATE score in predicting these outcomes highlights its importance as a tool in surgical planning and decision-making.

## Introduction

Minimally invasive hepatobiliary surgery has been increasingly applied worldwide over the past years [1]. Patients receiving minimally invasive liver resections appear to have shorter postoperative hospital stay, lower blood loss and transfusion requirement, and fewer post-operative complications when compared to patients undergoing open surgery [2]. However, the majority of liver resections is still performed by an open approach in most countries [3]. One reason for this sluggish implementation is that minimally invasive hepatobiliary surgery can be technically challenging.

In order to predict technical difficulty of planned liver resections preoperatively, a difficulty scoring system has been developed for laparoscopic liver resections. Thus, patient selection according to the technical difficulty and the skill level of the surgeon can be effectively performed based on this difficulty scoring system referred to as the IWATE criteria [4]. The IWATE criteria consist of a classification system respecting six preoperative aspects and grading the difficulty of liver resections into *low*, *intermediate*, *advanced*, and *expert.* The IWATE criteria have been validated in different cohorts and have been shown to effectively predict intraoperative and postoperative outcomes of laparoscopic hepatectomies [5].

Recently, there has been a rapid increase of robotic liver surgery, which may overcome the above-mentioned challenges of minimally invasive liver surgery due to its increased magnification, three-dimensional view, improved range of motion, and tremor control.

The objective of the current study is to assess the outcomes of a robotic liver surgery program at a German tertiary center during the implementation phase in terms of morbidity and mortality. As a secondary aim, the validity of the IWATE criteria in predicting these outcomes will be assessed.

## Methods

A robotic surgery program was started at our institution in October 2020. Only one of the surgeons had previous experience with robotic surgery, whereas the rest of the team had no previous experience. Based on the prospective database of the Interdisciplinary Robotic Center at Ulm University Hospital, the first 100 consecutive robotic liver surgery cases were identified. The prospective robotic database is registered at the German Clinical Trials Register (DRKS00024946) and all patients were followed-up for 90 days postoperatively. All cases were discussed within a multidisciplinary board preoperatively (infectiology board for cases of echinococcosis and tumor board for malignant cases), consisting of specialists from departments comprising surgeons, medical oncologists, radiologists, gastroenterologists, infectiologists, and pathologists. Two of the surgeons had some previous experience with minimally invasive liver surgery. However, liver surgery was usually performed by an open approach before implementation of the robotic program at our institution. During the study phase, there were no contemporary cases of laparoscopic liver resections. Patients, in whom vascular resections and reconstructions were anticipated, were primarily selected for open surgery. Apart of this, there were no formal selection criteria and during the course of the study, robotic surgery was chosen as the primary approach for nearly all other liver resections.

Demographic data, encompassing age, sex, body mass index (BMI), American Society of Anesthesiologists (ASA) score, and previous hepatic surgeries, were collected. Furthermore, details about the diagnosis and the surgical procedure including the aspects of the IWATE score were gathered. Apart from these baseline characteristics and surgical details of the patients, the following outcome measures were assessed: perioperative outcome parameters including estimated blood loss (ml), duration of surgery (min), postoperative length of hospital stay (days), morbidity according to the Clavien/Dindo classification, and 30-day as well as 90-day mortality [6]. Major morbidity was defined as any morbidity ≥ grade 3. Postoperative morbidity outcomes included surgical site infections (SSI) according to the definition of the Centers for Disease Control and Prevention (CDC) [7]. Furthermore, liver-specific complications comprising postoperative biliary leakage (BL), posthepatectomy liver failure (PHLF), and posthepatectomy hemorrhage (PHH) were assessed to the respective definitions of the International Study Group of Liver Surgery [8–10]. Furthermore, the comprehensive complication index (CCI) was calculated as a measure of the overall burden of postoperative complications [11].

### Surgical procedure

The DaVinci Xi Surgical System® (Intuitive Surgical Inc., Sunnyvale, CA, USA) was used for the surgical procedures. During surgery patients were positioned supine with legs apart and a slight anti-Trendelenburg position of 10–15° with a left tilt of 5–10°. Using a Veress needle at the left midclavicular line at the subcostal margin a pneumoperitoneum was established with an average pressure of 12–14 mmHg. Five trocars were utilized in total, where four robotic trocars with an 8-mm diameter were placed horizontally 2–3 cm above the umbilicus with spacing of approximately 8 cm apart from each other. Additionally, a 12-mm assistant trocar was positioned below trocars 2 and 3 in a triangular fashion. For major hepatectomies, another 5-mm trocar was placed below trocars 1 and 2. In contrast, patients undergoing atypical or anatomic resections of segments VII and/or VIII were placed in a left lateral position on a vacuum mattress. In these cases, the pneumoperitoneum was established by a puncture with the Veress needle at the right subcostal margin. The four robotic trocars were placed 2–3 cm below and parallel to the right subcostal margin approximately 8 cm apart from each other in these cases and a 12-mm assistant port was placed caudally between robotic trocars 2 and 3 in triangular fashion.

After completion of the trocar settings, a laparoscopic diagnostic exploration of the abdominal cavity was performed in all cases in order to rule out previously undetected intraabdominal pathologies. After adequate mobilization of the liver according to the respective procedure, an intraoperative ultrasound was performed in all cases in order to identify the lesions and liver vasculature and to the determine the resection plane. Indocyanine green fluorescence was utilized optionally to visualize major liver vessels, the biliary tree or segmental borders. A Glissonean approach was usually performed for major and anatomic resections.

Parenchymal transection during the surgical procedures involved the use of bipolar forceps and monopolar scissors or the SynchroSeal device (Intuitive Surgical Inc., Sunnyvale, CA, USA). Additionally, in some cases, especially major resections, a laparoscopic ultrasonic surgical aspirator was used. A Pfannenstiel incision was performed to retrieve the specimen within an extraction bag, except for patients with previous laparotomies, in which this site was favored.

In cases of simultaneous or exclusive radiofrequency ablation, the Cool-tip™ HF-ablation system (Medtronic GmbH, Meerbusch, Germany) was used.

Regarding perioperative management, an institutional fast-track protocol was applied, including early mobilization and early oral nutrition starting on the first postoperative day.

### Statistical analysis

A descriptive statistical analysis was performed, providing medians and interquartile ranges (IQR) for continuous variables and absolute frequencies and percentages for binary or categorical variables. Despite the descriptive analysis, outcome parameters were compared for the different categories of the IWATE criteria. For univariate analysis of categorical data, the Fisher’s exact test or Chi-square test was used. Considering a non-normal distribution of continuous variables, the Kruskal–Wallis or Mann–Whitney *U*-test was applied for the analysis of continuous variables. A *p*-value < 0.05 was considered statistically significant.

## Results

From November 2020 until January 2023 a total of 100 patients underwent robotic liver surgery at our institution, of which 41 (41%) were female and 59 (59%) were male patients. Median age was 65 years (IQR 13.5) and median BMI was 25.9 (IQR 5.1). Further baseline characteristics and underlying diagnoses are displayed in Table 1.

**Table 1:** Demographic data and clinical characteristics of the patients

	*N* = 100
Median age in years (IQR)	60.6 (13.5)
Median BMI in kg/m^2^ (IQR)	25.9 (5.1)
Gender	
Female	41 (41%)
Male	59 (59%)
ASA score	
I	2 (2%)
II	27 (27%)
III	68 (68%)
IV	3 (3%)
Diagnosis/histology	
Alveolar echinococcosis	24 (24%)
HCC^*^	22 (22%)
CRLM	22 (22%)
Biliary tract cancer^†^	13 (13%)
Benign/premalignant liver lesions	11 (11%)
Metastases other than CRLM	8 (8%)

*ASA*: American Society of Anesthesiologists; *BMI*: body mass index; *CRLM*: colorectal liver metastases; *HCC*: hepatocellular carcinoma; *IQR*: interquartile range; *SD*: standard deviation

^*^One case of fibrolamellar HCC and one case of mixed HCC/CCC

^†^Three cases of gall bladder cancer and 11 cases of intrahepatic cholangiocarcinoma

Major liver resections (≥ 3 segments) were performed in 17% of cases and anatomic liver resections in 47%, including 3 cases of fully robotic ALPPS and one hybrid ALPPS hepatectomies. However, the majority of resections were atypical resections (49%), followed by anatomical segmentectomies (17%) and left lateral sectionectomies (13%). In four cases, robotic-assisted radiofrequency ablations were performed without formal resection. The median IWATE score (excluding radiofrequency ablation only cases) was 6 (IQR 4), with low, intermediate, advanced, and expert resections accounting for 23 (24%), 41 (42.7%), 15 (15.6%), and 17 (17.7%) of resections, respectively (Table 2). Median duration of surgery was 180 (IQR 128.7) min. Median estimated blood loss was 300 (IQR 550) ml, and 22 (22%) patients received perioperative blood transfusions (Table 2).

**Table 2:** Operative details of robotic surgery

	*N* = 100
Major resection (≥ 3 segments)	
Yes	17 (17%)
No	83 (83%)
Anatomic resection	
Yes	47 (47%)
No	53 (53%)
Multivisceral resection	
Yes	6 (6%)
No	94 (94%)
Previous hepatic surgery	
Yes	13 (13%)
No	87 (87%)
Surgical procedure	
Radiofrequency ablation	4 (4%)
Atypical resection^*^	49 (49%)
Anatomical segmentectomy^†^	17 (17%)
Left lateral sectionectomy^‡^	13 (13%)
Left hemihepatectomy	2 (2%)
Right hemihepatectomy	11 (11%)
Fully robotic ALPPS^§^	3 (3%)
Hybrid ALPPS^||^	1 (1%)
IWATE level^¶^	
Low	23 (24%)
Intermediate	41 (42.7%)
Advanced	15 (15.6%)
Expert	17 (17.7%)
Median IWATE score (IQR)^¶^	6 (4–8)
Median duration of surgery in minutes (IQR)	180 (128.7)
Median estimated blood loss in milliliter (IQR)	300 (550)
Perioperative blood transfusions	22 (22%)

^*^Three cases with simultaneous radiofrequency ablation and one case with simultaneous biliodigestive anastomosis

^†^Eight cases with simultaneous atypical resection and one case with simultaneous radiofrequency ablation

^‡^Three cases with simultaneous atypical resection, one case with simultaneous hemicolectomy, one case with simultaneous distal gastrectomy and segmental resection of transverse colon, and one case with simultaneous cyst deroofing

^§^Two cases of atypical extended right hemihepatectomy and one case of right hemihepatectomy with segment I resection

^||^Extended left hemihepatectomy + atypical resections in right liver lobe; first step robotic and second step open surgery

^¶^IWATE level and score given only for 96 patients; four patients with radiofrequency ablation only excluded

The median length of postoperative hospital stay was 6 (IQR 4) days. Conversion to open surgery was performed in 6 (6%) cases for the following reasons: intraoperative bleeding tendency (*n* = 3), vascular infiltration (*n* = 2), and adhesions (*n* = 1). Ninety-day mortality occurred in 2 (2%) cases and overall morbidity was present in 21 (21%) patients with major morbidity (≥ grade III) in 13 (13%) of patients. The mean CCI was 7.8 (SD 18.9). Further morbidity results including liver-specific complications are listed in Table 3.

**Table 3:** Postoperative outcomes

	*N* = 100
90-day mortality	2 (2%)
90-day overall morbidity	21 (21%)
90-day major morbidity (≥ grade III)	13 (13%)
Mean CCI (SD)	7.8 (18.9)
Surgical site infection (SSI)	9 (9%)
Superficial incisional SSI	0 (0%)
Deep incisional SSI	0 (0%)
Organ/space SSI	9 (9%)
Pleural effusion	5 (5%)
Postoperative bile leakage	4 (4%)
Grade A	1 (1%)
Grade B	2 (2%)
Grade C	1 (1%)
Posthepatectomy hemorrhage (Grade A)	1 (1%)
Posthepatectomy liver failure	7 (7%)
Grade A	4 (4%)
Grade B	3 (3%)
Grade C	0 (0%)
Reintervention^*^	10 (10%)
Reoperation	3 (3%)
Rehospitalization	11 (11%)
Median length of postoperative hospital stay in days (IQR)	6 (4)

^*^Including percutaneous drainage, pleurocentesis, and endoscopic interventions

**Fig. 1:**
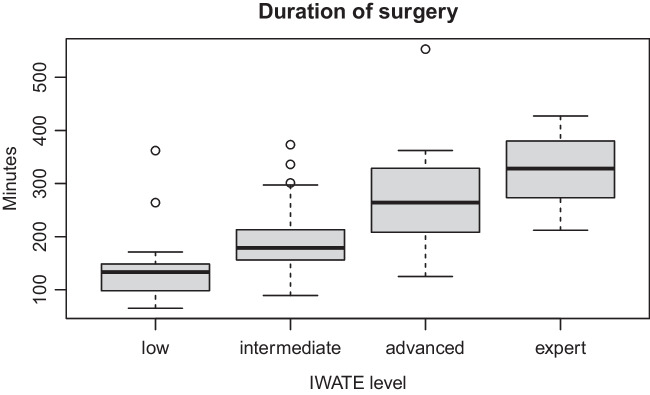
Boxplot displaying duration of surgery (minutes) for different IWATE categories (*p* < 0.001)

**Fig. 2:**
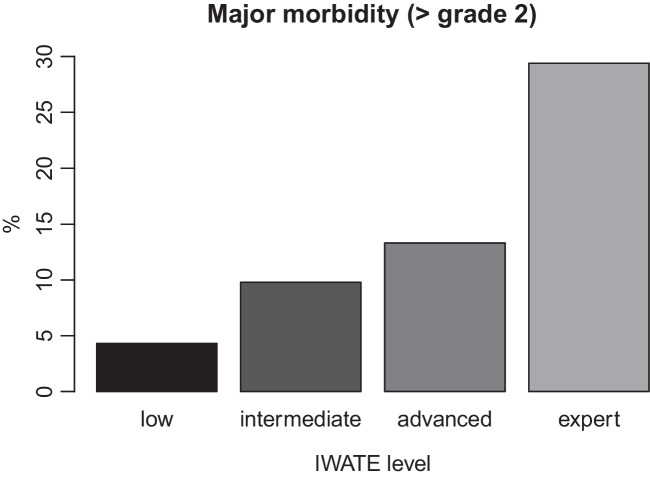
Correlation of IWATE criteria with Clavien-Dindo major morbidity (> grade 2) (*p* = 0.105)

The IWATE difficulty level demonstrated a statistically significant effect on duration of surgery (*p* < 0.001), estimated intraoperative blood loss (*p* < 0.001), and length of hospital stay (*p* < 0.001). Figure 1 displays these findings for duration of surgery. Furthermore, the IWATE level showed a significant influence on postoperative overall morbidity (*p* = 0.004). In particular there was increased morbidity in the expert group compared to all other difficulty groups. The rates of major morbidity showed a stepwise increase with increasing difficulty grades but without statistical significance. Rate of major morbidity for the different IWATE levels is displayed in Fig. 2. The CCI differed significantly only between the IWATE low and expert groups (*p* < 0.01). Table 4 demonstrates the outcome parameters for the different IWATE groups.

**Table 4:** Outcome parameter IWATE

	Low (*n* = 23)	Intermediate (*n* = 41)	Advanced (*n* = 15)	Expert (*n* = 17)	*p*-value
90-day overall morbidity^‡^	2 (8.7%)	7 (17.1%)	2 (13.3%)	9 (52.9%)	0.004^†^
90-day major morbidity (≥ grade III)	1 (4.3%)	4 (9.8%)	2 (13.3%)	5 (29.4%)	0.105^†^
Mean CCI (SD)^§^	1.5 (5.7)	7.8 (20.8)	3.6 (9.5)	19.9 (27.3)	0.004^*^
Median length of postoperative hospital stay in days (IQR)^∥^	4 (3)	5 (3)	7 (2.5)	8 (9)	< 0.001^*^
Median duration of surgery in minutes (IQR)^¶^	133 (50.5)	179 (57)	264 (120.5)	328 (107)	< 0.001^*^
Median estimated blood loss in milliliter (IQR)^**^	200 (300)	250 (300)	350 (650)	1200 (1000)	< 0.001^*^

^*^Kruskal–Wallis test

^†^Chi-square test

^‡^*p* < 0.05 for pairwise comparisons low vs. expert, intermediate vs. expert, and advanced vs. expert

^§^*p* = 0.001 for pairwise comparison low vs. expert (Wilcoxon rank sum test)

^∥^*p* < 0.05 for pairwise comparisons low vs. expert, intermediate vs. expert, and advanced vs. expert (Wilcoxon rank sum test)

^¶^*p* < 0.05 for all pairwise comparisons (Wilcoxon rank sum test)

^**^p < 0.05 for pairwise comparisons low vs. expert, intermediate vs. expert, and advanced vs. expert (Wilcoxon rank sum test)

## Discussion

The current cohort study demonstrates that robot-assisted surgery enables surgeons to perform liver surgery, including complex hepatobiliary procedures, by a minimally invasive approach with good outcomes. In a team of experienced hepatobiliary surgeons, an efficient robotic liver surgical program can be implemented within a reasonable timeframe even under the circumstances of the COVID-19 pandemic and without extensive previous experience in minimally invasive liver surgery. Furthermore, our results demonstrate that robotic liver surgery can be performed with good clinical outcomes even in the implementation phase. As an example, the postoperative 90-day mortality rate of 2% is below the threshold of in-hospital mortality of 7.5% from a recent nationwide analysis in Germany [12].

The IWATE difficulty grading system, which was actually developed for laparoscopic liver surgery, presents a helpful tool also for robotic liver surgery. The results of the current study demonstrate that the IWATE score is not only able to predict technical difficulty, e.g., in terms of duration of surgery and estimated blood loss, but also postoperative outcomes in terms of overall morbidity and the CCI. Our results are comparable to other reports on the implementation phase of robotic liver surgery regarding perioperative outcomes (duration of surgery and estimated blood loss) as well as rates of postoperative morbidity and mortality. In contrast to our findings, Luberice et al. found in their study that higher IWATE categories were associated with an increase in duration of surgery and estimated blood loss, but not with postoperative morbidity and mortality [13]. One of the reasons for the lack of an influence on postoperative outcomes may be that mortality and morbidity was surprisingly low with 0% in the expert group within their study. Similarly, Labadie et al. found a significant association of the different IWATE difficulty categories with duration of surgery, estimated blood loss, and also in length of hospital stay [14]. There was no formal analysis regarding the association of the IWATE difficulty levels with postoperative morbidity in this study, but in the expert group overall morbidity was 28% compared to 14% in the low and advanced difficulty groups and 10% in the intermediate group. This is in line with the findings of our study, in which overall morbidity was significantly increased in the expert group compared to all other IWATE difficulty levels. The differences between our current study and the previous studies [13, 14] regarding the prediction of postoperative outcomes by the IWATE score may be explained in part by the rather small sample sizes of the individual IWATE categories in all of the studies and by varying definitions of postoperative complications and their assessment (retrospective chart review vs. prospective assessment). Thus, it is yet not clear whether robotic surgery is able to diminish the impact of technical difficulty on postoperative outcomes as compared to laparoscopic liver surgery and further large studies are needed to clarify this issue.

There are several limitations to the current study. First, it was a cohort study of a single center. Thus, the results may not be generalizable to other sites. Second, this was a retrospective analysis with inherent risk of bias of this study design. However, the data quality and completeness of follow-up was high, because the analysis was based on a prospective database. Third, the sample size of the current study is limited, and a learning curve effect might have occurred, which has not been specifically evaluated. However, given the variety of procedures a formal learning curve analysis would not have been reasonable and the aim of this study was to transparently describe the implementation phase of a robotic hepatobiliary surgical program and evaluate the validity of the IWATE criteria in this regard.

In conclusion, in our study involving 100 robotic hepatectomy cases, we demonstrated that a robotic hepatobiliary surgical program can be implemented from scratch with good clinical outcomes and within a reasonable timeframe. Furthermore, we were able to demonstrate significant associations of the different IWATE difficulty levels with perioperative and postoperative outcomes. This finding emphasizes the usefulness of the IWATE criteria for robotic liver surgery in predicting the difficulty of resections and postoperative outcomes; therefore, the difficulty levels may aid in appropriate patient selection. Robotic surgery may enable surgeons to overcome the underutilization of minimally invasive liver surgery, but further prospective multicenter trials are necessary to validate these findings.

## Data Availability

A fully anonymized dataset can be obtained from the corresponding author upon reasonable request.
